# ROS-Mediated NLRP3 Inflammasome Activation in Brain, Heart, Kidney, and Testis Ischemia/Reperfusion Injury

**DOI:** 10.1155/2016/2183026

**Published:** 2016-04-05

**Authors:** Letteria Minutoli, Domenico Puzzolo, Mariagrazia Rinaldi, Natasha Irrera, Herbert Marini, Vincenzo Arcoraci, Alessandra Bitto, Giovanni Crea, Antonina Pisani, Francesco Squadrito, Vincenzo Trichilo, Daniele Bruschetta, Antonio Micali, Domenica Altavilla

**Affiliations:** ^1^Department of Clinical and Experimental Medicine, University of Messina, 98125 Messina, Italy; ^2^Department of Biomedical and Dental Sciences and Morphofunctional Imaging, University of Messina, 98125 Messina, Italy; ^3^Department of Human Pathology, University of Messina, 98125 Messina, Italy

## Abstract

Ischemia and reperfusion (I/R) causes a reduction in arterial blood supply to tissues, followed by the restoration of perfusion and consequent reoxygenation. The reestablishment of blood flow triggers further damage to the ischemic tissue through reactive oxygen species (ROS) accumulation, interference with cellular ion homeostasis, and inflammatory responses to cell death. In normal conditions, ROS mediate important beneficial responses. When their production is prolonged or elevated, harmful events are observed with peculiar cellular changes. In particular, during I/R, ROS stimulate tissue inflammation and induce NLRP3 inflammasome activation. The mechanisms underlying the activation of NLRP3 are several and not completely elucidated. It was recently shown that NLRP3 might sense directly the presence of ROS produced by normal or malfunctioning mitochondria or indirectly by other activators of NLRP3. Aim of the present review is to describe the current knowledge on the role of NLRP3 in some organs (brain, heart, kidney, and testis) after I/R injury, with particular regard to the role played by ROS in its activation. Furthermore, as no specific therapy for the prevention or treatment of the high mortality and morbidity associated with I/R is available, the state of the art of the development of novel therapeutic approaches is illustrated.

## 1. Introduction

The term ischemia and reperfusion (I/R) indicates a reduction of arterial blood supply to tissues followed by the restoration of perfusion and consequent reoxygenation [1]. In humans, ischemia, with the consequent fall in blood supply, is generally induced by the presence of an arterial embolus, which induces a severe tissue hypoxia in a coexistent inflammatory environment secondary to different risk factors, such as diabetes, hyperlipidemia, and aging. Experimental studies examining the mechanisms and the consequences of I/R use surgical methods to block specific vessels in otherwise healthy animals [2], therefore, these models are indicated to better understand the mechanisms involved in the injury induced by I/R.

The restoration of blood flow causes further damage to the ischemic tissue through neutrophil infiltration, reactive oxygen species (ROS) accumulation, deregulation of cellular ion homeostasis, and cell death with consequent inflammatory responses. Furthermore, in addition to local damage, I/R can also induce deleterious remote effects, resulting in the development of systemic inflammatory responses and multiple organ dysfunction syndrome [3].

Reperfusion is characterized by an early and a late phase, during which, due to reduced adenylate cyclase activity and intracellular cAMP levels, free radicals, such as ROS, are generated [4], and the mechanisms of cell death are triggered.

ROS are free radicals containing the oxygen atom, among which there are hydrogen peroxide (H_2_O_2_), superoxide anion (O_2_^∙−^), and hydroxyl radical (OH^•^). They originate mainly within the mitochondria, as a bioproduct of oxygen metabolism, but can also be generated by cellular enzymes, including lipoxygenase (LOX) and cyclooxygenase (COX) [5].

Under normal conditions, ROS have beneficial effects, as they regulate several important, physiological responses by redox-responsive signaling pathways. In fact, ROS control cellular growth, differentiation, and migration, regulate the vascular tone and cellular adhesion, contribute to the production of iNOS at transcriptional and posttranscriptional level by redox-dependent Nuclear Factor-*κ*B (NF-*κ*B) or mitogen activated protein kinases (MAPKs), and modulate immune response and control angiogenesis and apoptosis [5–7].

When ROS production is prolonged or elevated, detrimental events are observed with peculiar changes in cellular proteins, lipids, and ribonucleic acids, leading to cell dysfunction or death. Several enzymes with antioxidant activity are involved in neutralizing ROS: among them, superoxide dismutase (SOD), *γ*-glutamyltransferase (GGT), glutathione (GSH), glutathione reductase (GSSG-Rd), glutathione peroxidase (GSH-Px), glutathione S-transferase (GST), and catalase (CAT) are included [8]. All these antioxidant systems are differently expressed in various organs. In fact, the GSH system shows moderate concentrations in kidney, heart, and brain, while GGT, GSSG-Rd, SOD, CAT, and GSH-Px are highest in the kidney, when compared to the brain [9], the heart [10], and the testis [10]. In addition, the antioxidant systems are differentially expressed in adult organs and in various embryonic stages [10].

**Figure 1:**
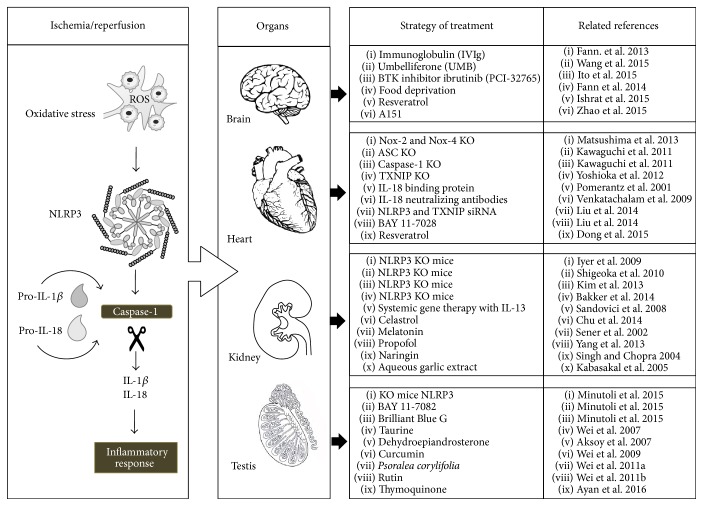
Schematic representation of ischemia/reperfusion injury and of potential therapeutic strategies to modulate oxidative stress and/or related NLRP3 activation.

The imbalance between ROS formation and the detoxifying action of these oxidizing radicals induces a cellular condition, called oxidative stress [11]. In particular, ROS, during I/R, promote tissue inflammation and activate immune response through NLRP3 inflammasome [11] (Figure 1).

The innate immune system is based on pattern-recognition receptors (PRRs) to sense pathogenic microbes and other endogenous or exogenous pathogens, such as pathogen-associated molecular patterns (PAMPs) and damage-associated molecular patterns (DAMPs). These immune activators initiate and regulate innate immune responses when identified by several classes of PRRs, including Toll-like receptors (TLRs), RIG-I-like receptors (RLRs), Nod-like receptors (NLRs), AIM2-like receptors (ALRs), C-type lectin receptors (CLRs), and other DNA sensors [12, 13]. PRRs trigger the activation of specific signaling pathways, which induce the production of many proinflammatory cytokines and chemokines and present antigens to the adaptive immune system for long-lasting protection.

NLRs form a large protein family of intracellular sensors, the members of which share a conserved central nucleotide-binding, oligomerization domain (NOD), a leucine-rich repeat (LRR) region, and a variable N-terminal effector domain [14, 15]. The family members of inflammasome are numerous [16], but NLRP3 inflammasome is the best characterized.

In a series of experimental models* in vitro* [17, 18], it was shown that two different steps, controlled by two different mechanisms, are required for the activation of NLRP3. The former is driven at transcriptional level by NF-*κ*B, whose increased production in I/R injury is activated via TLR signaling through Myd88-dependent pathways, with the subsequent stimulation of interleukin- (IL-) 1*β* and IL-18 gene expression [19, 20]. The latter is induced at posttranscriptional level and consists in the activation of NLRP3, driven by many activators, such as ROS in I/R injury [21].

When activated, the NLRP3 inflammasome is formed by the NLRP3 protein belonging to the family of NLRs, by the adapter protein apoptosis-associated speck-like protein (ASC), and by the procaspase-1 [15]. The assembly of NLRP3 moves procaspase-1 molecules near enough to transform them into active fragments [21], so that the conversion of the immature proinflammatory cytokines IL-1*β* and IL-18 to their active forms is induced [22, 23]. These cytokines then initiate or amplify diverse downstream signaling pathways and drive proinflammatory processes [24], leading to cellular damage, such as autophagy and pyroptosis [22]. The former is a process of self-degradation of parts of the cells through sequestering of organelles or parts of the cytoplasm and subsequent fusion with lysosomes [25]. Pyroptosis is a programmed cell death with loss of the cellular membrane integrity, differently from apoptosis, associated with IL-1*β* and IL-18 secretion; for this reason, it is considered an inflammatory form of cell death [26]. NLRP3 activation is able to promote also the initiation and the progression of different autoimmune and autoinflammatory diseases, such as metabolic disorders, inflammatory bowel syndrome [15], obesity, and cognitive diseases [27].

The mechanisms driving the activation of NLRP3, that is, NLRP3 oligomerization, ASC recruitment, and caspase-1 activation, are generally classified as noncanonical and canonical. The noncanonical pathway involves caspase-4 and caspase-5 in human cells and caspase-11 in mice and is activated by the identification of cell wall ligands, such as LPS, from phagocytized bacteria [28]. The canonical pathway is based on the recognition of general cellular stress, such as the oxidative stress induced by I/R, bacterial toxins, and particulate substances [28]. Recent studies showed that NLRP3 might sense the presence of ROS produced into the same cell by normal or malfunctioning mitochondria [29]. In particular, it was proposed that increased ROS are sensed by a complex of TRX and TRX-interacting protein (TXNIP) and induce the dissociation of the complex. In normal cells, TXNIP is constitutively connected to and kept in the reduced state by the ubiquitous TRX [30]. Following an increase in cellular ROS concentration, this complex dissociates and TXNIP binds to the LRR region of NLRP3, leading to NLRP3 activation [31].

Given the growing evidence that NLRP3 inflammasome activation is involved in many systemic diseases [32, 33] and considering that conflicting data exist with respect to I/R injury, the present review was aimed to evaluate the current knowledge on the role of NLRP3 in some organs (brain, heart, kidney, and testis) after I/R injury, with particular regard to the role played by ROS in its activation. Furthermore, as no specific therapy exists for the prevention or treatment of the great mortality and morbidity associated with I/R, the state of the art of the development of therapeutic strategies was also evaluated.

## 2. ROS and NLRP3 in I/R Injury of the Brain

It is well known that reperfusion may exacerbate the brain injury initially caused by ischemia, producing an I/R injury [3]. Among the various underlying mechanisms of stroke, inflammation and oxidative stress are implicated in the pathogenesis of brain I/R, and an adequate regulation of inflammatory level may play a critical role in the prevention and treatment of stroke [34]. In the last years, the role of inflammasomes, particularly NLRP3, has been recognized in postischemic inflammation after stroke. Since the inflammasome initiates inflammation, modulation of NLRP3 inflammasome can regulate inflammatory response. However, from a molecular point of view, NLRP3 inflammasome pathway can be activated by a variety of molecular signaling systems in I/R brain and many mechanisms have not been fully defined yet.

IL-1*β* is a crucial contributor to excitotoxic and ischemic brain injury and central inflammatory responses [35, 36]. Specifically, this cytokine is produced during a central nervous system disease or after a brain injury by macrophages or microglial cells [37] and molecular mediators as mitochondria-derived ROS and lysosomal protease cathepsin B are necessary for the microglial cell production of interleukin-1*β* [38].

So far, NLRP3 inflammasome dependent responses* in vitro* are linked to an initial stimulus by a pathogen or damage-associated molecular pattern (DAMP) [18, 39, 40]. Recently, it has been shown that DAMPs could induce inflammatory responses through the production of IL-6 and chemokine (C-X-C motif) ligand 1 (CXCL1) and the release of cathepsin B, in the absence of any bacterial infection or products in cultured mouse mixed glia [41]. Savage and coworkers [41] also revealed that IL-1 production contributed to increase of IL-6 and CXCL1 levels following cerebral ischemia by middle cerebral artery occlusion in mice, confirming that DAMPs amplify brain inflammation by directly stimulating production of glial derived inflammatory mediators. In this context, acute phase protein serum amyloid A could act as a priming stimulus on glial cells [41]. Together, these data add new helpful information on molecular pathways of brain inflammation and/or ischemic brain damage that involve glial cells and NLRP3 inflammasome-activating DAMPs, in the absence of cell priming and in the presence of a relevant endogenous priming stimulus, leading thus to new interesting perspectives from a therapeutic point of view.

So far, a number of recent studies focused on pathophysiological mechanism and/or potential therapeutic strategy to modulate inflammatory and oxidative stress pathways involving NLRP3 inflammasome in stroke brain damage (Figure 1).

Indeed, the expression pattern of the NLRP3 inflammasome in primary cortical neurons subjected to simulated ischemia, in a mouse model of focal ischemic stroke, and in brain tissue samples from stroke patients has been described [42]. Interestingly, these authors showed that intravenous immunoglobulin treatment protected brain cells in experimental stroke models by a mechanism involving suppression of NLRP3 activity [42].

Likewise, another interesting paper of Fann et al. [43] reported that 16 hours of food deprivation, daily, for 4 months, can attenuate the inflammatory response and tissue damage following focal ischemic stroke in mice through inhibition of NLRP3 inflammasome activity. Moreover, NLRP3 deficiency ameliorates neurovascular damage in experimental ischemic stroke [44].

Bruton's tyrosine kinase (BTK) is a tyrosine kinase involved in NLRP3 inflammasome activation leading, in turn, to caspase-1 activation and mature IL-1*β* production in the ischemic brain [45]. Interestingly, Ito and coworkers [45] showed that ibrutinib (PCI-32765), a potent BTK inhibitor [46], suppresses NLRP3 inflammasome signal in a focal brain I/R model. Specifically, ibrutinib exerts neuroprotective effects through the suppression of IL-1*β* maturation in infiltrating macrophages and neutrophils in the infarcted area.

As mentioned before, oxidative stress is a crucial hallmark in the pathophysiology of brain damage after stroke. To this purpose, it has been indicated that ROS are proximal signals for NLRP3 inflammasome activation in inflammatory diseases [47]. Experimental evidences demonstrate that an increase in ROS concentration following cellular stress leads to TRX oxidation, TXNIP recruitment of NLRP3, and consequent NLRP3 activation [48]. In fact, the action of curcumin in the hippocampus subjected to glutamate neurotoxicity was recently demonstrated [48];* in vitro* and* in vivo* results showed that curcumin attenuated glutamate neurotoxicity by inhibiting endoplasmic reticulum stress-associated TXNIP/NLRP3 activation via the regulation of AMP-Activated Protein Kinase and thereby protected the hippocampus from ischemic insult [48].

Umbelliferone (UMB) is a natural compound belonging to the coumarin family with antioxidant properties. As a matter of fact, in an interesting paper of Wang et al. [49], pretreatment with UMB ameliorated the neurological outcomes, the infarct volume, and the brain edema in a rat model of focal cerebral ischemia induced by middle cerebral artery I/R. These results indicate that UMB exerts partly these neuroprotective effects through the inhibition of TXNIP/NLRP3 signal. Moreover, another study suggests that TXNIP plays a critical role in acute ischemic stroke because it is directly linked to redox imbalance and NLRP3 activation. The latter also suggests the importance of the antioxidant effect of resveratrol on the TRX/TXNIP system in mice subjected to embolic middle cerebral artery occlusion [50].

Recently, it has been shown that A151, a synthetic oligodeoxynucleotide containing multiple telemeric TTAGGG motifs, reduces ischemic brain damage and NLRP3 mRNA levels in Stroke-Prone Spontaneously Hypertensive rats submitted to permanent middle cerebral artery occlusion [51].

Collectively, these data strongly confirmed that NLRP3 represents a potential therapeutic target in the management of ischemic stroke. Therefore, an appropriate treatment of brain I/R injury with compounds showing anti-inflammatory/antioxidant activity and targeting different and complex molecular pathways, also including NLRP3 activation, remains a big therapeutic challenge in translational medicine. In fact, it is nowadays difficult to establish the appropriate timing about their use and/or the duration of treatment to counteract the brain parenchymal damage in the setting of I/R injury.

## 3. ROS and NLRP3 in I/R Injury of the Heart

Myocardial I/R injury is a pathological process causing cardiac cells necrosis and apoptosis, in particular when the coronary perfusion is restored [52].

A large number of studies have demonstrated an increased ROS formation either during the ischemic phase or during the reperfusion period. Excessive ROS induce cell injury by disrupting cellular signaling transduction, activating inflammation factors, and inducing lipid peroxidation [53] and even cell death [54]. Additionally, ROS have been identified as an important NLRP3 inflammasome activator in cardiac diseases [55].

When activated, NLRP3 forms an inflammasome complex with the adaptor molecule ASC, thus controlling the activation of caspase-1; the latter cleaves pro-IL-1*β* and pro-IL-18 into the biologically active forms, thus initiating the sterile inflammatory disease [56]. In a recent work [52], KO mice for NLRP3 showed larger infarct size than wild type, so a protective role of NLRP3 inflammasome was suggested. However, the results of the study were critically discussed and disproved by Toldo et al. [57].

IL-1*β* and ASC are key players in I/R injury as they are important and early mediators of the inflammatory response in myocardial I/R injury (Figure 1). In fact, ASC deletion and IL-1*β* inhibition protect the myocardium from I/R injury in mice [58, 59]. In myocardial I/R injury, an important role is played also by IL-18, whose expression is stimulated in cardiomyocytes by ROS [60]. Specifically, IL-18 may induce myocardial injury through the induction of inflammation, increased apoptosis, and changes in calcium overload [61]. The administration of human myocardiocytes of IL-18 binding protein, a potent inhibitor of IL-18 activity, improved contractile function [58] and showed a protective role in the cardiac inflammatory response against I/R injury in mice [62]. Similarly, the treatment of mice with IL-18 neutralizing antibodies prior to I/R injury reduced the infarct size [60].

As to the mechanisms by which ROS induce inflammasome activation in the heart, many doubts are still present. TXNIP is ubiquitously expressed in normal tissues and is an endogenous inhibitor of TRX as, when directly connected, it prevents TRX activity to scavenge ROS [63]. Therefore, TXNIP KO mice showed a protection from I/R injury in cardiomyocytes [64]. However, the mechanism by which TXNIP mediates cardiac injury is still not clear. Recently, in myocardial I/R injury a role of TXNIP in the activation of the NLRP3 inflammasome was proposed through a direct interaction in cardiac microvascular endothelial cells, after intramyocardial administration of NLRP3 and TXNIP siRNA and of BAY 11-7028, an inflammasome inhibitor [63] (Figure 1).

Many clinical studies examined the role of antioxidants in ROS-mediated I/R injury through the administration of antioxidant drugs after thrombolysis, but the results were not positive in reducing infarct size or enhancing heart function [65]. On the contrary, in mice KO for NADPH oxidases (Nox) 2 and 4, contributing to part of ROS production during I/R injury, a reduction in ROS production and a decrease of the infarct size were observed after I/R [66]. However, it was suggested that a total inhibition of Nox is not positive as it is involved in the physiological and beneficial production of ROS [66]. Among antioxidant drugs, resveratrol protects the heart during I/R injury by inhibition of NALP3 inflammasome and ROS production [67] (Figure 1).

## 4. ROS and NLRP3 in I/R Injury of the Kidney

I/R injury is one of the common causes of acute renal failure, thus playing a significant impact on patient morbidity and mortality [33]. Kidneys are particularly vulnerable to ischemia; therefore, I/R injury may cause early graft rejection in renal transplantation and induce structural damage after suprarenal aneurysm repair, renal artery reconstruction, contrast agent-induced nephropathy, cardiac arrest, and shock [68]. Even if ischemia initiates a complex, organized series of events, resulting in damage and death of renal cells due to the dramatic decrease in oxygen and nutrition, reperfusion, though essential for tissue survival, determines an exacerbation of tissue injury and a profound inflammatory response, leading to renal dysfunction [1]. Increasing ischemia time worsens the histological changes, which are particularly severe at 24–72 hours after reperfusion.

Epithelial cells, particularly those of the S3 segment of the proximal tubule in the outer renal medulla, are particularly exposed to both ischemia and reperfusion phases of I/R injury, which can lead to acute tubular necrosis [69, 70]; on the contrary, glomerular vessels degeneration was described only in the reperfusion phase [69]. Histopathological evaluation revealed the presence of extensive vascular dilatation, slight interstitial edema, tubular dilatation, tubular cell swelling, brush border, and nuclear loss [71]. Furthermore, I/R injury induces an early infiltration of inflammatory cells, mainly neutrophils [72], in addition to the rapid tubular necrosis determining an acute renal dysfunction [73]. However, it was observed that the less I/R-sensitive cells of the distal tubules might have a protective role, leading to reepithelialization of the injured tubules and preventing the progression to chronic kidney disease [74].

There is now substantial evidence that ROS and NLRP3 inflammasome have a key role, even if not coincident, in the primary mechanism through which I/R induces the above indicated kidney damage.

In fact, Iyer et al. [75] showed that nonlethal renal I/R injury resulted in a significant upregulation of NLRP3 gene expression, which was accompanied by pronounced acute tubular necrosis. It was suggested that specific forms of cellular injury result in the release of viable mitochondria into the extracellular space, triggering the activation of the NLRP3 inflammasome, in part through the release of ATP.

Furthermore, upregulation of NLRP3 activates caspase-1 and, subsequently, IL-1*β* and IL-18; in addition, caspase-1 induces pyroptosis, a proinflammatory form of programmed cell death, characterized by pores in the plasma membrane at early time points [33].

Several pharmacological approaches were proposed to reduce the functional and morphological damage induced by I/R in kidney. In particular, the pathways of oxidative stress and NLRP3 inflammasome were examined by the administration of antioxidant drugs or by the study of NLRP3 knock-out (KO) animals (Figure 1).

As to the drugs acting on the ROS production and/or scavenging in the kidney, the pretreatment with the pineal hormone melatonin had a protective effect against oxidative damage caused by free radicals in a number of models both* in vivo* and* in vitro* of I/R injury: in particular, when administered prior to ischemia and immediately before the reperfusion, melatonin reduced the renal structural changes and limited the neutrophils infiltration [69].

Similar results were obtained from the pretreatment with naringin [76], aqueous garlic extract [77], rutin [78], propofol [79], celastrol [71], and allopurinol and apocynin, administered alone or together [80]; all these therapeutic strategies were based on the antioxidant properties of these substances, which are able to positively act on the exaggerated inflammatory responses and tissue damage dependent on the free radicals production and the inflammatory cells infiltration [53].

The pretreatment with modified adenovirus expressing IL-13, which is known to display antioxidant properties, diminished renal tubulointerstitial damage and inflammation induced by I/R [81].

Important data on the mechanisms involved in the I/R of kidney were obtained from the study of KO animals. In fact, in IL-18 KO mice, a reduced tubular damage and higher protection against I/R injury [82] were observed. In NLRP3 KO mice, an increased proliferation of tubular epithelial cells was observed, thus indicating that NLRP3 is detrimental to the repair response after the reperfusion phase of the I/R injury [83]. Similarly, in NLRP3 KO mice following I/R injury, an increased protection from lethal ischemic injury [56] and a reduced tubular necrosis and apoptosis, with consequent repopulation of the tubular epithelium, were observed after the reperfusion phase [84]. On the contrary, when ASC KO mice were examined, the protection from lethal ischemic injury was less pronounced [32, 75] and the tubular necrosis and apoptosis were higher and similar to those observed in WT mice [84]. It was evident that, at least in the kidney, the NLRP3 protein, the essential component of the inflammasome, may play an independent role in injury signaling, different from that of the other components of the inflammasome (ASC, caspase-1).

## 5. ROS and NLRP3 in I/R Injury of the Testis

Testis torsion is a testicular lesion typical of the pediatric population and it is representative of the I/R injury observed in other organs [1, 85]. Under these circumstances, the testis produces several proinflammatory cytokines, whose increased levels can be considered an indirect evidence of tissue inflammation [86].

In normal conditions, enzymatic antioxidant defense systems, such as SOD, GPx, and CAT, protect testicular somatic and germinal cells from free radical damage. On the contrary, malondialdehyde (MDA) is an important indicator of lipid peroxidation induced by ROS [87].

I/R induces early tissue injuries, such as reactive oxygen species (ROS) generation [4], and a damaged barrier function of endothelial cells, resulting from reduced adenylate cyclase activity and intracellular cAMP levels. In particular, during I/R, nucleotides in the form of ATP stimulate tissue inflammation and trigger NLRP3 inflammasome [88]. This pathological cascade causes a decreased number of germ cells induced by an enhanced apoptosis, the vacuolization of the seminiferous epithelium, a reduced number of spermatozoa, and a recruitment of neutrophils [89]; at a later stage, testicular atrophy and impaired spermatogenesis are observed [90–92].

In order to prevent testicular I/R injury, the effects of different substances with antioxidant activities and the role of NLRP3 inflammasome have been investigated (Figure 1).

Among antioxidant substances, lipoic acid (LA) has ROS scavenging and metal chelating ability and regenerates endogenous antioxidants, such as glutathione and vitamins E and C [87]. The pretreatment with LA induced an increase of SOD and GPx activity, reduced MDA levels, and abated cellular damage.

Similar results have been obtained by the pre- or posttreatment with different antioxidant pharmacological approaches, including taurine [93], dehydroepiandrosterone [94], curcumin [95],* Psoralea corylifolia* [96], rutin [97], thymoquinone [98], and apocynin [99].

As to the role of NLRP3 inflammasome, specific inhibitors, such as BAY 11-7082 [100] and Brilliant Blue G (BBG) [101], are able to inhibit its effects in a testicular I/R model [102]. In fact, BAY 11-7082, an I-*κ*B kinase-*β* inhibitor, and BBG, blocking the membrane-bound purinergic P2X7 receptor, showed a significant reduction of IL-1*β* and IL-18 mRNA expression, blunted caspase-1 and caspase-3 expression, minor histological damage, low TUNEL activity, and preserved spermatogenesis, indicating a selectively reduced NLRP3 inflammasome activity [102]. It was also observed that NLRP3 KO mice responded to I/R insult with a lower activation of the inflammatory and apoptosis cascade than WT animals.

Therefore, NLRP3 can be considered an interesting target for innovative drugs aimed at treating I/R injury after testicular torsion.

## 6. Conclusions

The restoration of blood flow as soon as possible is without any doubt the primary therapeutic approach to ischemia, even if reperfusion, although essential to restore oxygen and nutrients supply and to remove potentially harmful products of cellular metabolism, can induce further pathological processes in the same organ and tissue injuries in other organs.

The main points to be stressed as a conclusion of this review can be summarized as follows:

- ROS have beneficial effects, as they regulate several important, physiological responses by redox-responsive signaling pathways, but, during I/R, they promote tissue inflammation and activate immune response through different pathways, including NLRP3 inflammasome.
- Not all organs demonstrate equal sensitivity to ischemia [2], the brain being the most sensitive to reductions in its blood supply; all organs demonstrate similar sensitivity to reperfusion injury, whose key events are inflammation and oxidative stress.
- The effects of ROS-mediated NLRP3 inflammasome activation in course of I/R injury in other experimental models are strongly suggested; in fact, by the examination of the existing literature, no data on limb, intestine, and ovary ischemia are present.
- In some of the already available experimental models, such as the heart, the testis, and the kidney, further studies using KO mice (NLRP3 or ASC) or antagonists of the NLRP3 cascade are needed to better understand the physiopathological events during I/R injury.

In conclusion, despite the increased literature of the past decade, a definite comprehension of the role of NLRP3 inflammasome in the host responses to different danger signals is still lacking. A detailed examination of the molecular mechanisms driving NLRP3 inflammasome transcription, assembly, and activation is needed to elucidate these processes in the different organs. This experimental approach, given the role of NLRP3 in several sterile inflammatory diseases, could be the basis for the design and elaboration of novel NLRP3 inflammasome inhibitors, thus avoiding the exclusive use of substances with antioxidant activity in patients with ischemic damage.

## Abbreviations

I/R: Ischemia and reperfusion
ROS: Reactive oxygen species
NLRP3: NOD-like receptor family pyrin domain containing 3
LOX: Lipoxygenase
COX: Cyclooxygenase
TRX: Thioredoxin
CAT: Catalase
PRRs: Pattern-recognition receptors
PAMPs: Pathogen-associated molecular patterns
DAMPs: Damage-associated molecular patterns
TLRs: Toll-like receptors
RLRs: RIG-I-like receptors
NF-*κ*B: Nuclear Factor kappa-light-chain-enhancer of activated B cells
NLRs: Nod-like receptors
ALRs: AIM2-like receptors
CLRs: C-type lectin receptors
IL-1*β*: Interleukin-1*β*
IL-18: Interleukin-18
TXNIP: TRX-interacting protein
SOD: Superoxide dismutase
GPx: Glutathione peroxidase
MDA: Malondialdehyde
KO: Knock-out
BBG: Brilliant Blue G.
